# Reconfigurable all-dielectric metamaterial frequency selective surface based on high-permittivity ceramics

**DOI:** 10.1038/srep24178

**Published:** 2016-04-07

**Authors:** Liyang Li, Jun Wang, Jiafu Wang, Hua Ma, Hongliang Du, Jieqiu Zhang, Shaobo Qu, Zhuo Xu

**Affiliations:** 1 College of Science, Air Force Engineering University, Xi’an 710051, Shaanxi, China; 2 Electronic Materials Research Laboratory, Xi’an Jiaotong University, Xi’an 710049, Shaanxi, China

## Abstract

Based on effective medium theory and dielectric resonator theory, we propose the design of reconfigurable all-dielectric metamaterial frequency selective surfaces (FSSs) using high-permittivity ceramics. The FSS is composed of ceramic resonators with different band stop responses under front and side incidences. By mechanically tuning the orientation of the ceramic resonators, reconfigurable electromagnetic (EM) responses between two adjacent stopbands can be achieved. The two broad stopbands originate from the first two resonant modes of the ceramic resonators. As an example, a reconfigurable FSS composed of cross-shaped ceramic resonators is demonstrated. Both numerical and experimental results show that the FSS can switch between two consecutive stopbands in 3.55–4.60 GHz and 4.54–4.94 GHz. The design method can be readily extended to the design of FSSs in other frequencies for high-power applications.

Frequency selective surface (FSS) is a kind of periodic structures that can be used to select incoming electromagnetic waves with different operating frequencies, polarizations, and incident angles. Due to this filtering property, FSS can be used in many applications. Conventional FSS usually consists of a 2-D periodic array of slots/apertures etched out of a conducting plate or of conducting patches/strips printed on a dielectric substrate^1^,^2^,^3^,^4^. However, the metallic parts of the conventional FSSs are prone to breakdown, oxidization and corrosion, especially in high-power and high-temperature applications. Therefore, it is imperative to develop new types of FSSs without using metallic structures. Instead of metallic patches, high-permittivity ceramic particles are employed as the elementary unit cell to fabricate all-dielectric metamaterial FSSs.

The study of the application of dielectric materials in filters may date back to many decades ago. Bertoni^5^,^6^ *et al*. studied frequency-selective reflection and transmission of a periodically modulated dielectric layer. They found that at millimeter frequencies, dielectric layers have advantages over metallic screens in low absorption loss. Park^7^,^8^,^9^ *et al*. used dielectric grating waveguide as filters. These works inspired researchers to develop all-dielectric periodic structures in filtering applications. Magnusson^10^,^11^,^12^,^13^,^14^,^15^,^16^,^17^,^18^,^19^,^20^ *et al*. designed many all-dielectric structures or filters based on guided-mode resonance effects in all-dielectric waveguide gratings at microwave frequencies and optical frequencies. Recently, Barton *et al*. designed an all-dielectric FSS based on guided-mode resonance for high-power applications at microwave frequencies^21^, and they further developed a methodology for the design of all-dielectric FSS using fast Fourier transforms and genetic algorithm optimizations^22^. In Barton’s work, all-dielectric FSS device was successfully tested at a peak power of 1.7 GW/m^2^, indicating that all-dielectric FSS can operate normally under high power^21^. The device was also tested in the passband at a high pulsed microwave with a power of 45.26 MW/m^2^ and no damage was observed^22^. This shows that all-dielectric FSS have advantage over conventional metallic FSS under high power. Bossard^23^,^24^,^25^,^26^,^27^,^28^ *et al*. also did impressive work in designing all-dielectric FSS at infrared or terahertz frequency using liquid crystal and single inhomogeneous dielectric layer. With the development of all-dielectric FSSs, tunable FSS^26^,^27^,^28^,^29^,^30^,^31^ were further proposed and realized. These tunable FSSs are mostly based on liquid crystals, and yet some of them still need metallic screens.

Zhao^32^ *et al*. and Lepetit^33^ *et al*. used dielectric resonators, instead of metallic patterns, to design all-dielectric metamaterials. They pointed out that metamaterials fabricated with metallic inclusions possess conduction losses and anisotropy, which limit their electromagnetic properties. In the framework of metamaterials, Li^34^ *et al*. proposed all-dielectric metamaterial FSS at microwave frequencies based on high-permittivity ceramic resonators. With this framework, the effective impedance of all-dielectric FSSs can be designed by tuning the effective relative permittivity and permeability. The ability of changing these values at will is depending on how to inspire the desired resonant modes in the dielectric resonators. With this ability, an alternative method can be used to design tunable or reconfigurable FSS.

In this paper, we proposed the design of reconfigurable all-dielectric metamaterial frequency selective surfaces (FSSs) using high-permittivity ceramics, without using any metallic parts. Due to the high-permittivity, the ceramic particles are sub-wavelength in size. Thus, the macroscopic electromagnetic properties of the proposed FSS can be described equivalently by effective permittivity and permeability. When the orientation of ceramic resonators is changed, the EM response of the FSS can be switched between two adjacent bands. To verify the design method, a reconfigurable FSS composed of cross-shaped resonators was designed, fabricated and measured. The resonant frequencies of the ceramic particles can be tuned by adjusting the geometrical parameters, so as to make the first two resonant modes properly spaced and to form two adjacent stopbands. The FSS can switch between a 0.4 GHz stopband in 4.54–4.94 GHz and a 1.0 GHz stopband in 3.55–4.60 GHz. Both the simulation and experiment results verify the band stop property of the designed FSS. Since such FSSs use high-permittivity ceramic particles as the unit cell, ohmic losses and low breakdown voltages inherent to metallic structures are avoided. Hence, such FSSs own great advantages in high-temperature and high-power applications. This kind of reconfigurable FSS can achieve two adjacent stop bands by rotating one structure. It can be used in RCS reduction techniques and reconfigurable antenna systems, etc.

## Methods

### The basic principle of designing all-dielectric metamaterial FSS

At microwave regime, high-permittivity ceramics can be used to design all-dielectric metamaterial. All-dielectric metamaterials work in the framework of effective medium theory and dielectric resonant theory. By tuning geometrical shapes, size parameters and the relative permittivity, different resonant modes can be excited in the dielectric particles at designed frequencies. In this case, they worked as sub-wavelength dielectric resonators. By inserting sub-wavelength high-permittivity dielectric resonators into the low-permittivity matrix, the macroscopic electromagnetic response can be described by effective relative permittivity and permeability. With the ability of tuning the resonant modes, the effective relative permittivity and permeability can be changed at will at the designed frequencies. For example, specific resonant modes can be designed to obtain metamaterial with negative permittivity and/or permeability.

The idea of designing metamaterials can be used to design FSSs. Generally speaking, the transmission characteristics of FSSs are mainly determined by the effective impedance of the FSS, which is, matching in the pass band while mismatching in the stop band. The effective impedance is determined by the effective permeability and permittivity of the proposed dielectric FSS. For example, the impedance of metamaterial FSS with single-negative electromagnetic parameter is difficult to match with free space, and then a stopband may emerge. The impedance of metamaterial FSS with double negative electromagnetic parameters can be designed close to the impedance of free space, leading to a band pass response. With the ability of tuning effective relative permittivity and permeability at will, the effective impedance of all-dielectric FSSs can be designed in the desired frequencies. Thus, the transmission characteristics of all-dielectric FSSs can be adjusted via the resonant modes of the dielectric resonators. To design FSSs with passband or stopband in the considered frequencies, the tuning of effective electromagnetic parameters at will is the key point, which is the essential idea of metamaterial. Thus, in the framework of metamaterial, all-dielectric FSSs can be well designed.

### Model and simulation of the reconfigurable FSS

**Figure 1:**
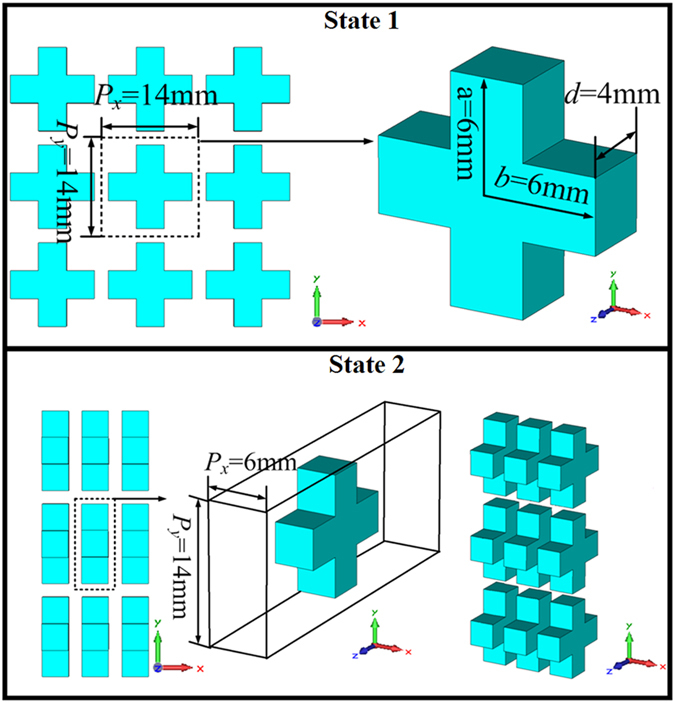
Unit cell of the all-dielectric reconfigurable FSS.

**Figure 2:**
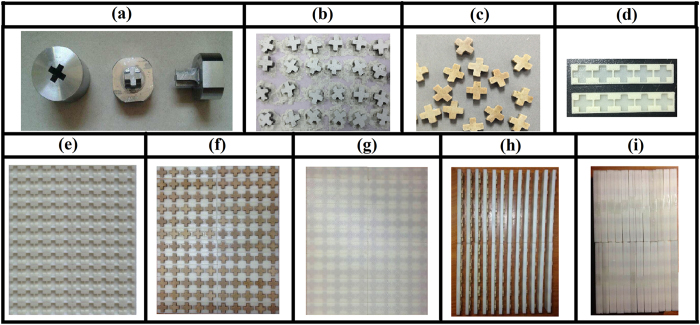
(**a**) Mould of the cross-shaped FSS. (**b**) Green-pressing of cross-shaped ceramics. (**c**) Sintering cross-shaped ceramics. (**d**) The pedestal part and roof cover of the supporting matrix. (**e**) The whole pedestal part of the supporting matrix. (**f**) State 1 cross-shaped ceramics array in the pedestal part without roof cover. (**g**) Sample of state 1. (**h**) State 2 cross-shaped ceramics array in the pedestal part without roof cover. (**i**) Sample of state 2.

**Figure 3:**
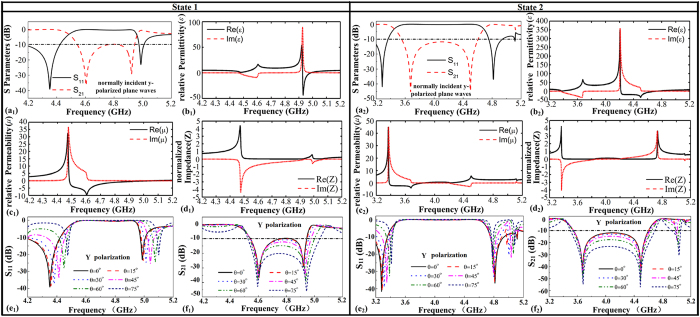
(**a**_**1**_**,a**_**2**_) Simulated transmission and reflection for state 1 and state 2 for normally incident y-polarized plane waves. (**b**_**1**_**,b**_**2**_) The relative permittivity for state 1 and state 2. (**c**_**1**_**,c**_**2**_) The relative permeability for state 1 and state 2. (**d**_**1**_**,d**_**2**_) The normalized impedance for state 1 and state 2. (**e**_**1**_**,e**_**2**_) Y polarization variation of S_11_ with the angle of incidence for state 1 and state 2. (**f**_**1**_**,f**_**2**_) Y polarization variation of S_21_ with the angle of incidence for state 1 and state 2.

The FSS consists of cross-shaped ceramic particles, as shown in Fig. 1. The relative permittivity and the loss tangent of the ceramics are ε_r_ = 110 and tanδ = 0.0015, respectively. As shown in Fig. 1, the length in x and y directions of the cross-shaped ceramic particle are with the same size of 12.0 mm. The thickness in the z direction of the cross-shaped ceramic particle is 4.0 mm. The unit cell occupies an area of 14.0 mm 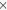 14.0 mm. In this case, it is defined as state 1. When the cross-shaped structure is rotated 90^o^ with respect to the y direction, the state is defined as state 2. The FSS made by cross-shaped ceramic is shown in Fig. 2. The EM responses under the two states were simulated using the frequency-domain solver of CST Microwave Studio. The simulated transmission/reflection spectra of the two states, as well as the extracted relative permittivity, relative permeability and normalized impedance are shown in Fig. 3.

### Sample preparation

The high-permittivity ceramics used in this paper are made of 0.7Ba_0.6_Sr_0.4_TiO_3_ –0.3La(Mg_0.5_Ti_0.5_)O_3_ ceramics, which is fabricated in solid-state sintering method. The production process is basically the same as that in our previous work^34^, except that the ball-milled hours, sintered temperature and holding time are a bit different. The permittivity of the ceramics is a little lower than the ceramics used in our previous work^34^. The main difficulty in making the sample is fabricating the cross-shaped ceramics in amounts. With this aim, the cross-shaped mould is prepared, as shown in Fig. 2(a). Polyvinyl Alcohol and dried 7:3 Ba_0.6_Sr_0.4_TiO_3_/La(Mg_0.5_Ti_0.5_)O_3_ powder was put into the mould and made into cross shape, as shown in the Fig. 2(b). Then, we sintered it to ceramics as shown in Fig. 2(c). 3D printer was used to fabricate a cross-shaped supporting matrix to package the cross-shaped ceramics. We make 5 cross-shaped ceramics in a group for the sake of easy assemble. The supporting matrix has two parts. One is the pedestal part, as shown in the bottom of Fig. 2(d). The other one is roof cover, as shown in the top of Fig. 2(d). The array of pedestal parts is shown in Fig. 2(e). Figure 2(f,g) show, respectively, state 1 and state 2 of the cross-shaped ceramics array in the pedestal part without the roof cover. Figure 2(g,i) are the final sample of state 1 and state 2, respectively. Then the samples shown in Fig. 2(g,i) were measured using vector network analyzer (VNA).

## Discussion

Figure 3(a1,a2) present the simulated transmission/reflection spectra of the two states for normally incident y-polarized plane waves. For state 1, there are two resonant dips at about 4.60 GHz and 4.92 GHz. For state 2, there are also two resonant dips at about 3.68 GHz and 4.50 GHz, which indicate that the resonances of the two states are different. Due to the resonances, for state 1, the transmission is below −10 dB in 4.54–4.94 GHz and the transmission is below −10 dB in 3.55–4.60 GHz for state 2, forming two adjacent stop-bands.

The planar period of the reconfigurable all-dielectric FSS is 14.0 × 14.0 mm and 6.0 × 14.0 mm. Considering that the central operating frequency of the FSS is at about 4.7 and 4 GHz, the unit cell size of the FSS is small enough compared to the operating wavelength. Therefore, the FSS can be described by the effective medium theory. The extracted relative permittivity and permeability of the two states are presented in Fig. 3(b1,c1,b2,c2). It can be found from Fig. 3 that strong resonances occur both for the relative permittivity and permeability. As shown in Fig. 3(b1,b2), the imaginary part of relative permittivity is positive around 4.9 GHz and 4.2–4.5 GHz, respectively. This means the magnetic energy is larger than the electric energy. It should be noted that this phenomenon usually accompany with electric resonance. With increasing frequencies, the relative permittivity achieve maximum firstly, and then drops to the minimum. This feature of the relative permittivity curve always occurs in electric resonance. As shown in Fig. 3(c1,c2), the imaginary part of relative permeability is positive around 4.5–4.6 GHz and 3.4–3.7 GHz, respectively. This means the electric energy is larger than the magnetic energy. The case that electric energy is larger than magnetic energy usually occurs in magnetic resonance. The feature that the relative permeability achieve maximum firstly, and then drops to the minimum with increasing frequencies always occurs in magnetic resonance.

The normalized impedance shown in Fig. 3(d1,d2) can explain why the stopband forms. At 4.2 GHz (d_1_) and 3.2 GHz (d_2_), the normalized impedance is 1, so the state 1 and state 2 are transparent to EM waves at 4.2 GHz and 3.2 GHz, respectively, as shown in Fig. 3(a1,a2). As illustrated in Fig. 3(c1,c2), the real part of relative permeability is negative and the real part of relative permittivity is positive at 4.5–4.9 GHz and 3.4–4.2 GHz, respectively. When the magnetic resonance occurs, the impedance matching becomes worse, resulting in reduced transmission. At about 4.6 and 3.7 GHz, the relative permeability reaches its negative maximum, whereas the relative permittivity is positive. These lead to extreme impedance mismatch. Thus the first transmission dip of the two states appears in Fig. 3(a1,a2). At a small area around 4.9 GHz in Fig. 3(b1) and 4.2–4.6 GHz in Fig. 3(b2), the real part of relative permittivity is negative and the real part of relative permeability is positive. At 4.9 GHz in Fig. 3(c1) and 4.5 GHz in Fig. 3(c2), the relative permittivity reaches its negative maximum, whereas the relative permeability restores positive. This leads to the second transmission dips in Fig. 3(a1,a2). In between the two resonant dips, as shown in Fig. 3(d1,d2), the real part of normalized impedance is close to 0 in 4.5–4.9 GHz and 3.4–4.7 GHz, respectively, leading to two consecutive stopbands.

Figure 3(e1,e2,f1,f2) show the variation of S_11_ and S_21_ curves when the incident angle of y-polarized waves changes from 0^o^ to 75^o^. The variations show that as the incident angle becomes larger, the operating band become wider and the band stop performance becomes better. This indicates that the FSS has good angle stability.

**Figure 4:**
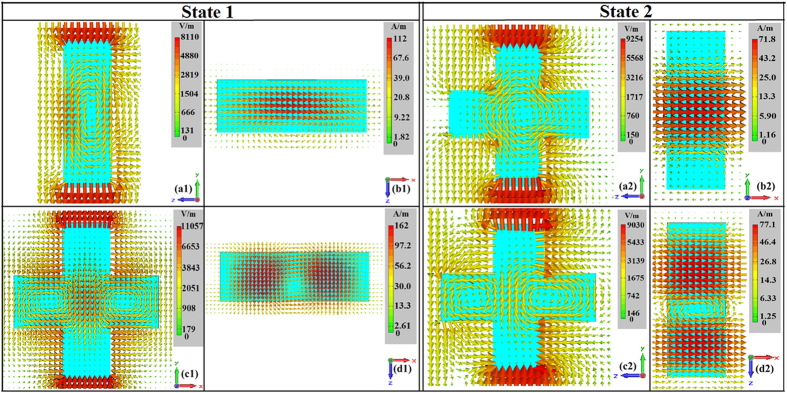
The distributions of electric fields and magnetic fields. (**a**_**1**_) Electric fields at 4.6 GHz. (**a**_**2**_) Electric fields at 3.68 GHz. (**b**_**1**_) Magnetic field at 4.6 GHz. (**b**_**2**_) Magnetic field at 3.68 GHz. (c_1_) Electric field at 4.92 GHz. (**c**_**2**_) Electric field at 4.5 GHz. (**d**_**1**_) Magnetic field at 4.92 GHz. (**d**_**2**_) Magnetic field at 4.5 GHz.

The physical mechanism of the FSS is further analyzed using the dielectric resonator theory^35^,^36^,^37^,^38^. The rectangular high-permittivity ceramic particle can be treated as a dielectric resonator^34^. The distributions of the electric and magnetic fields of the FSS under the two states at their resonant frequencies are shown in Fig. 4. Figure 4(a1,b1) show that the electric field loop is formed in y-o-z plane, equivalent to a magnetic dipole at 4.6 GHz. This is similar to 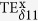 mode^34^ of the rectangular dielectric resonator, where the super-script x means the electric field is transverse with respect to x direction and 0 < δ < 1. Figure 4(c1,d1) show that the two electric field loops with opposite orientations are formed in x-o-y plane, equivalent to an electric dipole at 4.92 GHz. This is similar to 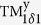 mode of the dielectric resonator. As is well-known, the electromagnetic response of a dipole is typically Lorentz-type. Thus, Lorentz-type resonance occurs for both the relative permeability and permittivity, as shown in Fig. 3(b1,c1). The negative relative permeability at 4.6 GHz indicates a magnetic resonance, also accompanies by an anti-resonance^39^ of the relative permittivity near 4.6 GHz. Similarly, the second resonance at 4.92 GHz is an electric resonance. The resonant modes of state 2 are nearly the same as state 1. We can see from Fig. 4(a2,b2), the electric field loop is formed in y-o-z plane, equivalent to a magnetic dipole at 3.68 GHz. This is similar to 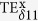 mode. Figure 4(c2,d2) show that the two electric field loops with opposite orientations are formed in y-o-z plane, equivalent to an electric dipole at 4.5 GHz. This is similar to 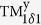 mode. Therefore, Lorentz-type resonance occurs for both the relative permeability and permittivity, as shown in Fig. 3(b2,c2). A negative resonance of the relative permeability and an anti-resonance of the relative permittivity near 3.68 GHz indicate that the resonance at 3.68 GHz is a magnetic resonance. The second resonance at 4.5 GHz is an electric resonance.

## Results

**Figure 5:**
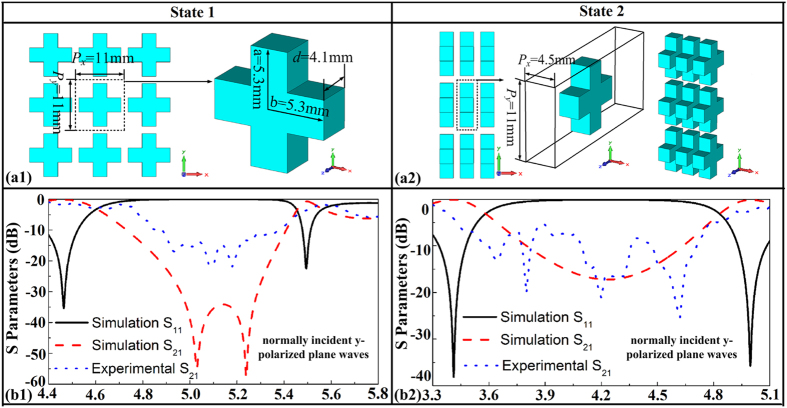
(**a**_**1**_) Unit cell of the re-designed reconfigurable all-dielectric with state 1. (**a**_**2**_) Unit cell of the re-designed reconfigurable all-dielectric with state 2. (**b**_**1**_) Adjusted simulation and experimental S parameters of the state 1 for normally incident y-polarized plane waves. (**b**_**2**_) Adjusted simulation and experimental S parameters of the state 2 for normally incident y-polarized plane waves.

The experimental results are shown in the Fig. 5(b1,b2). Figure 5(b1) gives the S_21_ of state 1 and Fig. 5(b2) gives the S_21_ of state 2. The experimental results are measured for normally incident y-polarized plane waves. It can be found that the working bands of the two states are moving to higher frequency, compared with the simulated results (Fig. 3(a1,a2)). Nevertheless, the reconfigurable characteristic is unchanged. This blue-shift phenomenon is due to the fact that ceramics shrink after sintering. The size of the cross-shaped mould is the same as the cross-shaped ceramics mentioned in Fig. 2. After a large number of fabrications, we found that the shrinking percentage of the 0.7Ba_0.6_Sr_0.4_TiO_3_–0.3La(Mg_0.5_Ti_0.5_)O_3_ ceramics is about 88%, so the resonator size we actually used is 88% of the mould size. Because the thickness of the ceramics depends on the amount of powders put into the mould, it’s in fact quite difficult to control. After our measurement, the thickness of the sintered cross-shaped ceramics is around 4.1 mm. That is to say, deviation was introduced when we used the mould to fabricate the green-pressing. What’s more, 3D printer also introduces errors. In order to match the supporting matrix and the cross-shaped ceramics, we have to magnify the size of the packaging. Errors in making the supporting matrix lead to the period of the cross-shaped ceramics deviated from the simulated one.

Considering the deviations in the fabrication process, the simulated model was adjusted according to practical cases. The arrangement unit cell of state 1 and state 2 are shown in the Fig. 5(a1,a2). Redesigned S parameters of state 1 and state 2 are shown in Fig. 5(b1,b2). These results are also simulated for normally incident y-polarized plane waves. There are tiny transmissions ripples in the experimental S_21_ curves, which are generated due to the fabrication errors in preparing ceramic resonators. The permittivity for hundreds of cross-shaped ceramics may not in the same value due to the unavoidable fluctuation of sintering temperature. The sintered shrinkage of the cross-shaped ceramics, the thickness of the green-pressing, the size of supporting matrix, the location and orientation of the cross-shaped ceramics in supporting matrix may have little deviation from the designed values. Thus, the measured results have some slight difference to the simulated one. Although there are some ripples appear in the experimental S_21_ curves, the frequency region of band stop and reconfigurable characteristic are kept. The experiment results are qualitatively in accordance with the simulated one. A more precise experiment can be achieved by utilizing high-precision processing conditions. Despite the deviations, the experiment still can verify the design of reconfigurable all-dielectric metamaterial FSS.

**Figure 6:**
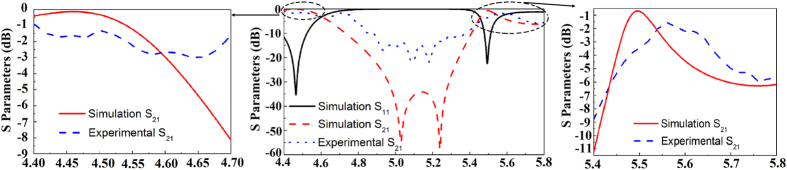
Detail areas of state 1.

**Figure 7:**
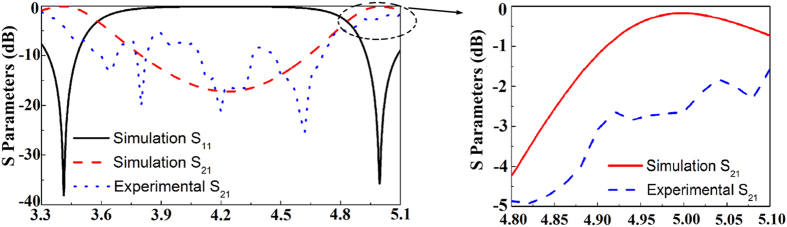
Detail area of state 2.

In order to show more detail of simulated and experimental results, the insertion loss of FSS in the passband are shown in Fig. 6 for state 1 and in Fig. 7 for state 2. For state 1 in 4.4–4.7 GHz, although the simulation result is not the same as experimental result, their average values are almost the same, along with the similar trends. In 5.4–5.8 GHz, the passband at about 5.5 GHz is very narrow and the peak value is between −0.5 dB and −1 dB. The peak of experimental result is red-shifted by about 0.1 GHz compared with the simulation results. After the narrow passband, it will have a stopband again. From experimental result, it can be seen that this trends is very close to the simulation one. As for state 2, the average value of experimental result in 4.8–5.1 GHz is about 1 dB lower than the simulation result.

Since band-stop responses are considered, low-loss response is not the most important feature in our manuscript. Nevertheless, to evaluate the low-loss response, it must be noted that low-loss do not mean low insertion loss. Since both the reflection and transmission must be considered simultaneously in the FSS design, the dielectric loss can be calculated by 1 − |S_11_|^2^ − |S_21_|^2^, which is different to the insertion loss in the passband (1 − |S_21_|^2^). Therefore, for example, at f = 5.454 GHz of simulated state 1, the dielectric loss is only −18.721 dB, demonstrating a low loss response. While the insertion loss at the same frequency reaches up to −5.090 dB.

**Figure 8:**
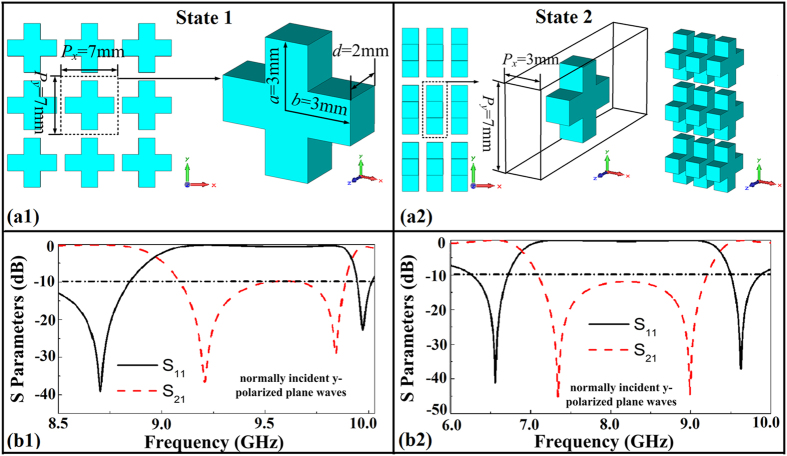
Reconfigurable FSS in other frequency achieved by scaling down the geometrical parameters.

For the fabricated sample, the impedance matching becomes worse due to the fabrication precision of the cross, leading to high insertion loss in the passband but enhanced reflection in the stopband. As for the stopband level, the difference between −60 dB, −40 dB, −20 dB is just the difference between 0.001, 0.01, 0.1. These errors are in toleration for engineering applications. It is vain to pursue the perfect match in the existing fabrication technology. Although these annoying errors are difficult to eliminate, the results and the trends are approximate to the simulation results, the rationality of the design method can be verified. Due to the scalability of the design, the method can be readily extended to the design of FSSs at other frequencies. For example, if the 3D sizes of the unit cell is reduced to 1/2 the original, the central operating frequency doubles, as shown in Fig. 8. The S parameters are acquired for normally incident y-polarized plane waves.

In conclusion, we propose the design of reconfigurable all-dielectric metamaterial FSS using high-permittivity ceramics. A cross-shaped reconfigurable all-dielectric metamaterial FSS is demonstrated in 3.55–4.94 GHz, which can switch between two consecutive stopbands under two states. The band stop characteristic can be tuned by adjusting the configuration of the resonators. This kind of reconfigurable FSSs can achieve two adjacent frequency selective characteristics with one structure. What’s more, such all-dielectric FSSs own important applications especially in high-temperature and high-power environments. By simply scaling the geometrical parameters, the method can be used to design FSSs at other frequencies.

## Additional Information

**How to cite this article**: Li, L. *et al*. Reconfigurable all-dielectric metamaterial frequency selective surface based on high-permittivity ceramics. *Sci. Rep*. **6**, 24178; doi: 10.1038/srep24178 (2016).
